# Effects of Tabata-style functional training and cinnamon supplementation on army combat fitness test performance, serum irisin, and body composition in military cadets: a randomized controlled trial

**DOI:** 10.1080/15502783.2026.2743157

**Published:** 2026-10-07

**Authors:** Reza Sabzevari Rad, Marefat Siahkouhian

**Affiliations:** a Department of Exercise Physiology, Faculty of Educational Sciences and Psychology, University of Mohaghegh Ardabili, Ardabil, Iran

**Keywords:** Body composition, cinnamon, functional nutrition, high-intensity interval training, irisin, the army combat fitness test

## Abstract

**Aim:**

The study aimed to assess the effect of Tabata-style functional training with or without cinnamon supplementation on the performance of the Army Combat Fitness Test (ACFT), serum irisin levels, and body composition in cadets at military academies.

**Methods:**

In a randomized controlled design, 48 male cadets (22.8 ± 1.3 years) were allocated to 4 groups: Tabata group (*n *= 14), cinnamon group (*n *= 10), Tabata+cinnamon (*n *= 14), and control group (*n *= 10). This was a 6-week intervention. Tabata training was performed three times per week (8 × 20s work/10s rest; 87%–95% HRmax), and cinnamon (*Cinnamomum zeylanicum*) supplementation was administered three times per day (1.5g per day). The ACFT components, serum irisin, body fat percentage (BFP), visceral fat index (VFI), lean body mass (LBM), and body mass index (BMI) were evaluated before and after treatment.

**Results:**

The group × time interaction was significant for ACFT score, irisin, and body composition markers (*p *< 0.001). Bonferroni post hoc showed that ACFT test scores in both groups, COMB and TAB, improved significantly compared to the CON group (*p *< 0.05). Significant improvement was observed for groups COMB and TAB in 3 repetition maximum Deadlift, Standing Power Throw, Release Pushups, Sprint-Drag-Carry, Leg Tuck, and 2-Mile Run compared to CON group (*p *< 0.05). The most significant increase in serum irisin levels was observed in COMB group compared to the other groups (*p *< 0.001). All groups showed significant reductions in body weight, BMI, and VFI when compared to the control group (*p *< 0.05). Among the groups, TAB group had a remarkably lower BFP than the others (*p *< 0.001). Additionally, LBM increased significantly in all groups compared to the control group (*p *< 0.001).

**Conclusion:**

Tabata-style training combined with cinnamon supplementation markedly enhanced performance, body composition, and circulating irisin in young male military cadets, yielding significant improvements in functional capacity and demonstrating the advantage of pairing high-intensity exercise with targeted nutritional intervention.

## 1. Introduction

The military force of the army functions as a performance-oriented entity, necessitating a high level of combat readiness and operational capability to effectively navigate unpredictable and challenging military scenarios. To succeed, military personnel must embody a blend of strength, endurance, and agility, enabling them to tackle various demands – from transporting heavy equipment over extended distances to responding promptly in high-pressure situations. Recognizing diverse needs, there is a strong commitment to evidence-based strategies that effectively integrate physical training for a more holistic approach to well-being. Such approaches are carefully crafted to improve operational performance while also supporting the long-term health and well-being of combat personnel [1,2]. In this regard, high-intensity interval training (HIIT) has emerged as a specialized methodology within the broader fitness domain. HIIT involves various workout formats that alternate between intense exercise and rest or lower-intensity activity. Interval durations can range from seconds to minutes, and the work-to-rest ratio can be adjusted based on individual needs [3]. In contrast, Tabata training is widely regarded as among the most time-efficient high-intensity interval training (HIIT) methods. Its distinguished protocol consists of eight cycles of 20s of intense exercise interspersed with 10s of rest [4]. This regimen has been associated with improvements in maximal oxygen uptake (VO_2_max), elevation of the lactate threshold, stimulation of mitochondrial biogenesis, and enhanced capacity for fat oxidation, and often with a total session duration of less than 30 min [5–7]. This unique approach to high-intensity interval training (HIIT) may be particularly appealing to individuals who have already achieved a high level of fitness. Tabata has shown consistent and beneficial effects on both aerobic and anaerobic measures. The fact that Tabata training is especially effective and requires little equipment makes it particularly suitable for military use, where training time is limited, logistical problems are common, and physical fitness is of the utmost importance for the success of the mission [8,9].

The rising popularity of HIIT has led to a greater interest in naturally available supplements that may facilitate physiological adaptation to exercise. Previous research indicates that the prevalence of nutritional supplement usage is greater among military personnel compared to the civilian population [10]. Nonetheless, there exists a limited body of research exploring the impact of nutritional supplements on physical performance in tactical athletes or military individuals. In contrast, a more extensive array of studies has examined the effects of supplementation in competitive athletes [11]. This discrepancy complicates the generalization of exercise performance findings to military personnel. Therefore, it is imperative to investigate the efficacy, dosage, and safety of nutritional supplements within military and tactical populations, as both muscle performance and recovery outcomes are crucial for the effectiveness of warfighters. Cinnamomum zeylanicum (true cinnamon) is of interest for its anti-inflammatory, insulin-sensitizing, and lipoprotein-lowering properties [12,13]. The thermogenic and anti-obesogenic properties of cinnamon cinnamaldehyde and eugenol appear to be mediated by the modulation of glucose metabolism and differentiation of adipocytes by the mitochondrial pathways [14–16]. Some researchers have suggested that cinnamon supplementation may inhibit visceral fat accumulation, improve glycemic control, and improve cardiovascular health, making it a suitable addition to any exercise program [17,18].

Irisin is a protein derived from FNDC5 that is released during muscle contraction. It plays a role in promoting the browning of white adipose tissue, enhancing thermogenesis, and influencing glucose and lipid metabolism [19]. Preclinical studies indicate that irisin may improve peripheral insulin sensitivity by increasing glucose uptake and enhancing insulin signaling in both muscle and fat tissues. However, human studies have demonstrated inconsistent associations between circulating levels of irisin, physical exercise, body fat, and metabolic health. These discrepancies may stem from measurement variability and population differences. Furthermore, the receptor and signaling mechanisms associated with irisin remain inadequately understood. Consequently, there are significant gaps in translating these findings into clinical applications, highlighting the need for further multidisciplinary research to determine whether targeting irisin pathways could serve as an effective strategy for combating obesity and insulin resistance [20,21]. In addition to metabolic activity, changes in irisin levels following exercise training – especially in high-intensity modalities such as Tabata and speed interval training – have been reported as indicators of adaptation [22,23]. Recent research suggests that elevated circulating irisin levels in response to high-intensity interval training (HIIT) may occur independently or alongside the beneficial physiological and metabolic properties of medicinal plants such as cinnamon, which is recognized for its high polyphenol content. These complementary effects may positively influence overall metabolic and physiological outcomes [24,25].

To date, no randomized controlled trial has examined the impact of combining Tabata-style training with a phytochemical supplement, such as cinnamon, on Army Combat Fitness Test (ACFT) performance, irisin levels, and body composition. ACFT uses a range of assessments to evaluate various physical disciplines, including: (1) strength (3RM deadlift), (2) power (standing power throw), (3) muscular endurance (hand-release push-ups), (4) anaerobic capacity (sprint-drag-carry), (5) trunk and grip strength (leg tuck or plank), and (6) aerobic capacity (two-mile run). This holistic approach helps individuals understand their physical capabilities and work towards their fitness goals [26]. However, as far as we know, no randomized controlled trial has so far been published that investigates the effects of a combination of Tabata-style training with phytochemical supplements, e.g. cinnamon, on the ACFT performance, irisin levels, or body composition.

This study addresses a critical knowledge gap by examining the effects of 6 weeks of cinnamon supplementation and Tabata functional training, administered alone and in combination, on ACFT performance, serum irisin concentrations, and body composition in healthy military cadets. This is the first study to investigate these parameters in an integrated manner, both under controlled conditions and in environments that reflect military situations. These findings may provide valuable insights for military training procedures and functional food strategies aimed at enhancing combat readiness, metabolic resilience, and overall performance.

## 2. Materials and methods

### 2.1. Study design and participants

This randomized controlled trial (RCT) was done in 6 weeks at Tehran Military Training Academy in Iran. Forty-eight healthy male officer cadets (mean age 22.8 ± 1.3 years) were recruited to participate in the study through institutional announcements and screened to be eligible. The participants were recruited in accordance with the following inclusion criteria: age between 20 and 25 years; BMI between 18.5 and 27kg/m^2^; and the absence of any diagnosis of a metabolic, cardiovascular, or musculoskeletal pathology. Exclusion criteria: factors that could affect the study, such as smoking, consumption of dietary supplements, or use of medication with an impact on metabolism, were excluded, as well as regular high-intensity exercise within the last 3 months. The research was conducted using written informed consent and was approved by the Ethics Committee of the Military University at the request of the authors in accordance with the requirements of the Declaration of Helsinki (approval code: IR.IAU.ARDABIL.REC.1403.229).

Participants were randomly selected to 1 of 4 groups: Tabata training (TAB; *n *= 14), cinnamon supplementation (CIN; *n *= 10), mixed Tabata+cinnamon (COMB; *n *= 14), and control (CON; *n *= 10) by a computer-generated sequence. Stratification was used as an aptitude test at baseline BMI. All participants kept to their normal military diet and routine tasks during the study.

### 2.2. Intervention protocols

#### 2.2.1. Functional HIIT based on Tabata-style 20:10seconds

In the control group, the study participants engaged in their usual daily activities. The 6-week training program consisted of 24 sessions, held three times a week on Sundays, Tuesdays, and Thursdays from 5:00 PM to 6:00 PM. Each session lasted 60 min and included a 10-minute warm-up, a 40-minute Tabata workout, and a 10-minute cool-down. The participants in the TAB and COMB groups attended Tabata-style functional training 3 days per week, non-consecutively, under supervision. In each round, the participants had to perform high-intensity functional movements such as walking lunges, burpees, bicycle crunches, inchworm to push-ups, opposite toe touch, triceps dips, push-ups, side plank, and Y-W-T scapular exercises for 20 s. In between, there was a 10-second rest period. The exercises were changed every week to avoid any adaptation. The sessions were administered outside, and HRM (Polar® HRM) was measured to ensure a target intensity between 87% and 95% HRmax, in accordance with best practices in HIIT [27] (Figure 1).

**Figure 1.**
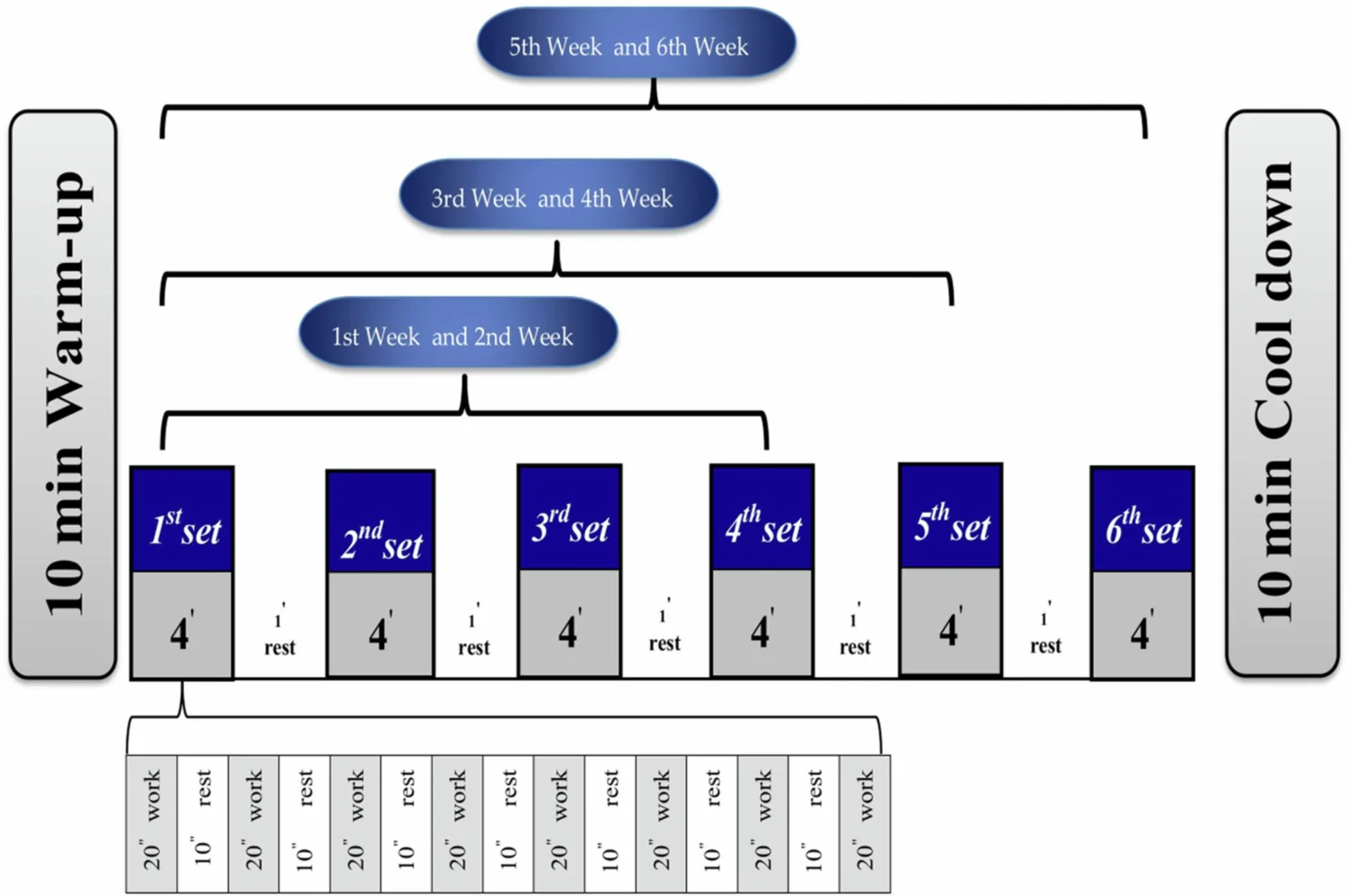
The functional HIIT based on Tabata-style 20:10s is illustrated, which consists of three phases: (1) warm-up, (2) main program, and (3) cool-down. The training intensity ranged from 87% to 95% of the maximum heart rate, as monitored with a Polar heart rate monitor. The Tabata training program was implemented over a 6-week period, with a frequency of three sessions per week. During the initial 2 weeks, male cadets engaged in four sets per session, with an incremental increase of one additional set every subsequent 2 weeks. Each set of the Tabata protocol was 4 min in duration, accompanied by a 1-minute rest interval. Within each 4-minute cycle, the male cadets performed 20s of high-intensity work followed by 10s of rest.

#### 2.2.2. Cinnamon supplementation

Over 6 weeks, participants in both the CIN and COMB groups added 1500 mg of cinnamon powder to their daily diet, dividing it into three doses taken with meals. The powder from the capsule of Cinnamomum zeylanicum was taken with 200–250 ml of water [28]. Cinnamon originating from pure herbal sources and containing phytochemical compounds has been tested by HPLC for batch consistency and for C. cassia contamination [29]. The form and dose have been chosen because previous studies have shown efficacy in improving metabolic and inflammatory outcomes without adverse effects [18,30].

### 2.3. Outcome measures

#### 2.3.1. Primary outcomes

The main and only outcome was the ACFT performance, which was measured both before and after intervention in accordance with the US Army recommendation [26]. The test components were: three repetitions of the max deadlift, standing power throw, hand release, sprint-drag-carry, leg tuck, and a 2-mile run. Each subject was given points for their performance in each of the six events using official standards, and a total score of composite points (0–600) was obtained. All participants in this test wore formal military uniforms, which included a t-shirt, boots, military cap, and military shirt and pants.

#### 2.3.2. Secondary outcomes

Blood samples (5mL) were obtained via antecubital venipuncture at baseline and after the 6-week intervention between 07:00 and 08:00h following a 10-hour overnight fast. The serum was separated by centrifugation and stored at −80 °C until analysis. Serum irisin concentrations were determined using a commercially available enzyme-linked immunosorbent assay (ELISA; BioVendor, Czech Republic). All samples were analyzed in duplicate according to the manufacturer’s instructions, and the mean value was used for statistical analysis. The intra-assay and inter-assay coefficients of variation were both<8%. To minimize inter-assay variability, samples from each participant were analyzed within the same assay batch.

Body composition was assessed using a multi-frequency bioelectrical impedance analyzer (InBody 380, South Korea), which has been validated for field-based assessment of body fat percentage, lean body mass percentage, visceral fat index, and body mass index. The device was calibrated according to the manufacturer’s guidelines prior to testing sessions. Measurements were conducted in the morning under standardized conditions. The participants were instructed to fast overnight, abstain from caffeine, alcohol, and strenuous physical activity for at least 12h, and void their bladder immediately prior to assessment. All measurements were performed barefoot, wearing light clothing, with participants standing upright and maintaining proper contact with the electrodes. Testing was performed in a thermoneutral environment by the same trained investigator to reduce measurement variability.

### 2.4. Diet

The participants were instructed to follow their usual dietary habits throughout the study. They submitted 3-day food records that included 2 weekdays and 1 weekend day, both at the beginning of the study and after an eight-week intervention. These records were then analyzed using Diet Analysis Plus version 10 to evaluate total energy intake and the contributions from proteins, fats, and carbohydrates [31].

### 2.5. Adherence and adverse events

Adherence to training and supplementation protocols exceeded 93% across all groups. No adverse events or gastrointestinal complaints were reported. The blinding of assessors was maintained throughout the study period.

### 2.6. Statistical analysis

The data were analyzed using SPSS version 25.0 (IBM Corp., Armonk, NY, USA). The Shapiro–Wilk test was used to determine normality. The pre-test scores of the corresponding outcome variables were employed as covariates in a multivariate analysis of covariance (MANCOVA) to evaluate differences in post-test outcomes between the groups. Bonferroni post hoc tests were used to compare the mean differences between the study groups. The effect sizes were determined using Cohen’s d (small: 0.2; medium: 0.5; and large: 0.8). A *p*-value of less than 0.05 was considered statistically significant. An a priori power analysis (GPower version 3.1) revealed that a total of 44 participants would be sufficient to achieve 80% power (f = 0.25) for a 5% alpha level for the main effect (group × time).

**Figure 2.**
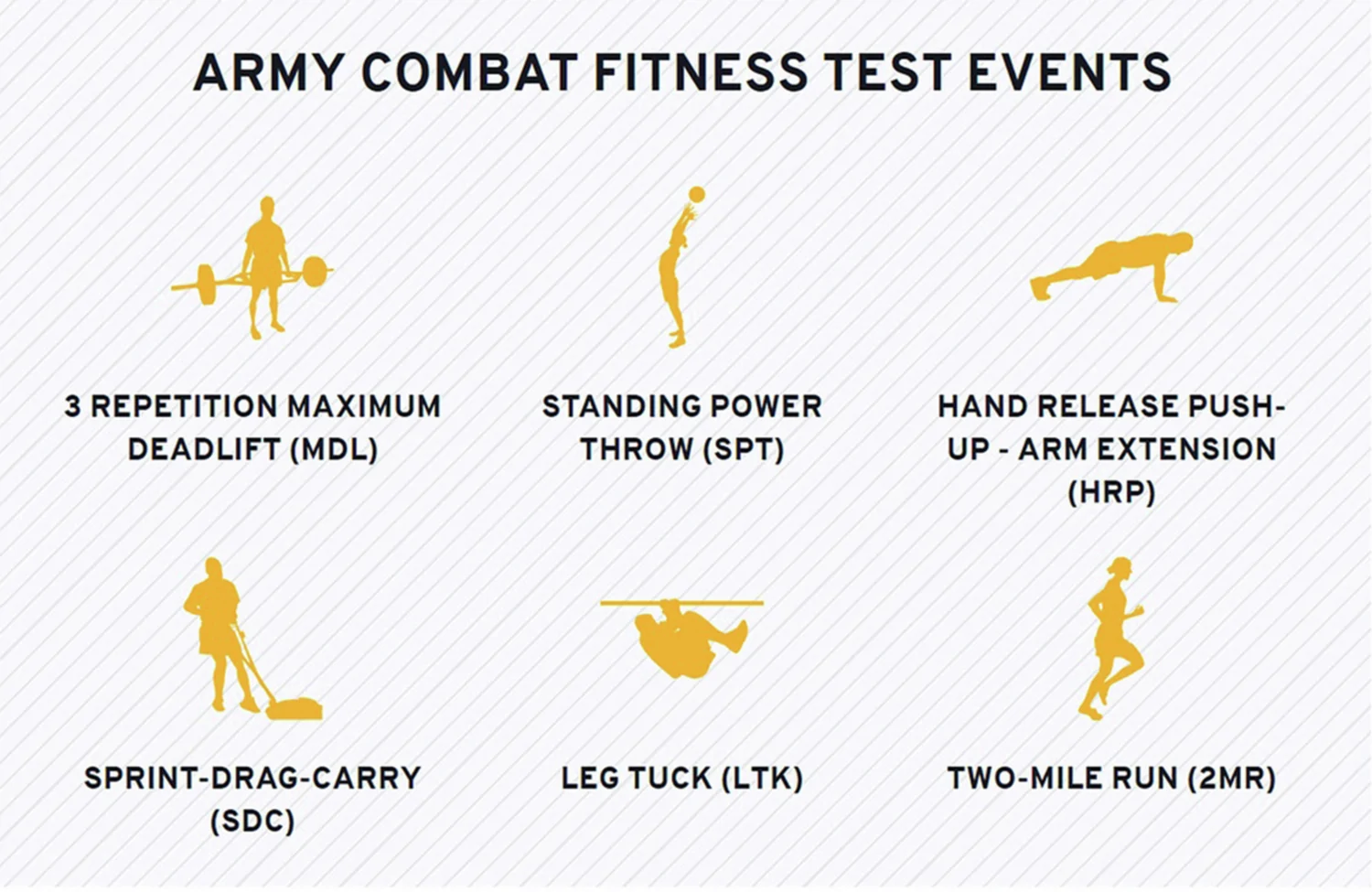
Army combat fitness test (ACFT) – scheme. This flowchart demonstrates the standardized order of six events of ACFT, which are aimed at measuring the main aspects of military physical readiness. Each task can stimulate a target-specific physiological area, and there are sufficiently spaced rest breaks to maximize test efficiency. (1) 3 repetition maximum deadlift: a method of evaluating maximal lower body strength, which is done by using a hexagonal bar. (2) Standing power throw: this is used to measure the explosive power through the overhead connection with the medicine balls being thrown backward. (3) Release Pushups – takes an upper-body muscular endurance measure by using a strict release movement. (4) Sprint-Drag-Carry: assesses the performance of anaerobic capacity and agility through five shuttle parts. (5) Leg Tuck: is a shoulder, arms, and core exercise that involves repeating the hanging leg tuck (or rather staying in the hanging leg tuck position) to test core strength. (6) 2-mile run: measures the aerobic energy-dense over an 18–21 min timed run on a flat surface. Rest intervals: 2–3 min between each test to allow some recovery, and 10 min rest between the core test (Leg Tuck/Plank) and the aerobic run.

## 3. Results

### 3.1. Participant and baseline characteristics

Of the 60 cadets initially screened, 48 met the inclusion criteria and were randomized into four groups (TAB = 14, CIN = 10, COMB = 14, CON = 10). Three participants withdrew due to unrelated medical leave (one each from TAB, COMB, and CON). Thus, 45 participants (TAB = 13, CIN = 10, COMB = 13, CON = 9) completed the study and were included in all the statistical analyses (Table 1).

**Table 1.** Baseline characteristics of the participants.

Measure	TAB (N = 13)	COMB (N = 13)	CIN (N = 10)	CON (N = 9)
**Anthropometrics and body composition**				
Age (y)	23.6 ± 0.4	22.5 ± 1.0	22.8 ± 1.3	21.6 ± 1.2
Height (cm)	175.2 ± 3.8	177 ± 3.7	180.4 ± 1.3	176.4 ± 5.0
BM (kg)	84.9 ± 4.9	78.2 ± 4.9	82.1 ± 3.9	80.9 ± 3.6
BMI (kg.m^−2^)	23.4 ± 1.7	23.4 ± 1.0	23.4 ± 2.2	22.6 ± 1.2
BFP (%)	21.6 ± 1.9	21.38 ± 1.5	21.8 ± 4.2	20.1 ± 1.3
VFI (cm^2^)	8.4 ± 1.44	10.5 ± 1.9	11.2 ± 2.8	9.4 ± 2.0
LBM (kg)	66.1 ± 3.4	59.95 ± 4.29	62.9 ± 3.0	64.9 ± 2.8
**Army combat fitness test**				
Deadlift (kg)	89 ± 17.44	88.5 ± 10.14	98 ± 27.6	105 ± 27.48
Standing Power Throw (meter)	7.1 ± 1.57	8.22 ± 1.72	7.6 ± 1.92	8.52 ± 2.68
Push-ups (reps)	32.2 ± 3.01	35 ± 6.46	40.4 ± 11.28	41 ± 11.52
Sprint-Drag-Carry (mm:ss)	1.62 ± .2	1.58 ± .22	1.44 ± .07	1.33 ± .42
Leg Tuck (reps)	2.4 ± .51	3.2 ± 2.14	5.6 ± 3.86	9.2 ± 6.74
2-mile run (mm:ss)	17.45 ± 1.09	17.13 ± 1.27	15.76 ± 1.05	17.13 ± 1.27
Total score	429.6 ± 7.0	443.6 ± 20.7	467.8 ± 35.1	480.6 ± 42.5
**Metabolic marker**				
Irisin (mcg/ml)	5.1 ± 0.5	4.8 ± 0.3	4.8 ± 0.3	5.1 ± 0.4

Abbreviations: y: Years, cm: centimeter, kg: kilograms, BM: body mass, BMI: body mass index, cm^2^: square centimeter, BFP: body fat percentage, VFI: Visceral fat index, LBM: lean body mass, rep: repetition, mcg/ml: micrograms per milliliter, TAB: Tabata training, COMB: Tabata training + cinnamon supplementation, CIN: cinnamon supplementation: CON: control.

### 3.2. Adherence and nutrient intakes

The training interventions for all groups had 100% adherence. There were no significant differences in average daily nutrient or energy intake, either within or between groups (*p *> 0.05; Table 2).

**Table 2.** Mean and standard deviation of dietary intake at pre- and post-intervention.

	Time	
	Pre	Post
**Energy (kcal. kg^−1^.d^−1^)**		
TAB	2467.0 ± 54.1	2489.2 ± 65.5
COMB	2396.5 ± 104.9	2434.1 ± 102.5
CIN	2456.0 ± 102.5	2431.7 ± 119.1
CON	2454.2 ± 94.6	2463.9 ± 105.3
**Protein (g.kg^−1^.d^−1^)**		
TAB	110.2 ± 6.9	111.0 ± 3.5
COMB	112.9 ± 10.3	109.1 ± 9.9
CIN	113.9 ± 7.9	118.1 ± 11.1
CON	109.5 ± 9.1	110.7 ± 13.5
**Carbohydrate (g.kg^−1^.d^−1^)**		
TAB	337.2 ± 15.2	346.3 ± 22.2
COMB	340.1 ± 9.8	330.1 ± 12.3
CIN	344.8 ± 14.9	338.0 ± 17.3
CON	337.9 ± 13.1	342.5 ± 18.6
**Fat (g.kg^−1^.d^−1^)**		
TAB	96.0 ± 9.7	96.4 ± 3.6
COMB	94.7 ± 7.1	93.3 ± 6.8
CIN	91.0 ± 3.7	94.8 ± 6.3
CON	91.1 ± 8.9	96.6 ± 12.7

Abbreviations: TAB: Tabata training, COMB: Tabata training + cinnamon supplementation, CIN: cinnamon supplementation, CON: control.

### 3.3. Effect sizes and clinical relevance

Cohen’s d effect sizes for the COMB group were large for ACFT total score (d = 1.42), serum irisin (d = 1.67), and body fat percentage (d = 1.11), indicating high practical significance. The TAB group yielded moderate to large effect sizes in similar domains, while the CIN group effects were small to moderate.

### 3.4. ACFT performance

The changes in ACFT performance are shown in Figure 3(A–G). A significant group × time interaction was observed for total ACFT score (F = 107.5, *p *< 0.001, η^2^ = 0.833). Post-hoc analysis revealed that both TAB and COMB groups showed significant improvements in total ACFT scores compared to the control group (TAB+8.6% and CIN 6.1%; *p *< 0.001; COMB:+12%, *p *< 0.001), indicating improved combat readiness. The COMB group outperformed the TAB group significantly (*p *= 0.001). Improvements were most pronounced in the sprint-drag-carry and 2-mile run components. The COMB group reduced 2-mile run records by 5.6% (from 17.13 ± 1.27 to 16.16 ± 1.29, *p *< 0.001), while the TAB group showed a 7.22% reduction (from 17.45 ± 1.09 to 16.19 ± 1.1, *p *< 0.001) and the CIN group showed a 2.66% reduction (from 15.76 ± 1.05 to 15.34 ± .91, *p *< 0.001). No significant changes were observed in the CON group (*p *> 0.05). Deadlift performance improved in the TAB (*p *< 0.05) and COMB (*p *< 0.001). The standing power throw increased significantly (TAB: +15%; *p *< 0.001; CIN: +29.7%; *p *< 0.001; COMB: +38.3%; *p *< 0.001). Push-ups improved notably (TAB: +26%, *p *< 0.001; COMB: +42%, *p *< 0.001). Sprint-Drag-Carry improved (TAB: +13.5%; *p *< 0.001, CIN: 7.63%, and COMB; 18.3%, *p *< 0.001). The leg tuck improved in all the intervention groups (*p *< 0.05 to *p *< 0.01) (Tables 3 and 4).

**Table 3.** Descriptive statistical analysis of body composition, army combat fitness test event scaled, total points, and serum irisin levels.

Measure	Intervention groups						Control group	
	TAB (N = 13)		CIN (N = 10)		COMB (N = 13)		CON (N = 9)	
	Pre (Mean ± SD)	Post (Mean ± SD)	Pre (Mean ± SD)	Post (Mean ± SD)	Pre (Mean ± SD)	Post (Mean ± SD)	Pre (Mean ± SD)	Post (Mean ± SD)
Body mass (kg)	84.9 ± 4.9	81.4 ± 4.9 ^#^	82.1 ± 3.9	79.9 ± 3.1^#^	78.2 ± 4.9	72.5 ± 4.9^$^	80.9 ± 3.6	81.6 ± 3.1
	∆=4.1 %		∆=2.6 %		∆=7.2 %		∆=0.8 %	
BMI (kg.m^−2^)	23.4 ± 1.7	20.6 ± 1.5*	23.4 ± 2.2	22.3 ± 2.2*	23.4 ± 1	19.54 ± 1*	22.6 ± 1.2	22.8 ± 1
	∆=11.9 %		∆=4.7 %		∆=16.4 %		∆=0.8 %	
BFP (%)	21.6 ± 1.9	18.7 ± 1.8^$^	21.8 ± 4.2	21 ± 4.2^$^	21.3 ± 1.5	17.3 ± 1.4^$^	20.1 ± 1.3	20.4 ± 1.4
	∆=13.4 %		∆=3.6 %		∆=18.7 %		∆=1.4 %	
VFI (cm^2^)	8.4 ± 1.4	6.7 ± 1.2^$^	11.2 ± 2.8	10.3 ± 2.9^$^	10.5 ± 1.9	7.6 ± 1.9^$^	9.4 ± 2	9.7 ± 2
	∆=20.2 %		∆=8.0 %		∆=27.6 %		∆=3.1 %	
LBM (kg)	66.1 ± 3.4	66.5 ± 3.0	62.9 ± 3.0	64.1 ± 3.1^$^	59.95 ± 4.29	61.5 ± 4.2^$^	64.9 ± 2.8	64.64 ± 3.67
	∆=0.6 %		∆=1.8 %		∆=2.5 %		∆=0.4 %	
3-RM Deadlift (kg)	89 ± 17.44	91 ± 18.7*	98 ± 27.6	102 ± 26.79^#^	88.5 ± 10.14	93 ± 21.36^#^	105 ± 27.48	107 ± 27.6
	∆=2.2 %		∆=4 %		∆=5 %		∆=1.9 %	
SPT (meter)	7.1 ± 1.57	8.23 ± 1.62^$^	7.6 ± 1.92	9.86 ± 1.25^$^	8.22 ± 1.72	11.37 ± 1.28^$^	8.52 ± 2.68	8.75 ± 2.78
	∆=15.9 %		∆=29.7 %		∆=38.3 %		∆=2.69 %	
Push-ups (reps)	32.2 ± 3.01	40.6 ± 1.83^$^	40.4 ± 11.28	42 ± 8.76	35 ± 6.46	49.7 ± 6.73^$^	41 ± 11.52	38.2 ± 9.96
	∆=26.0 %		∆=3.96 %		∆=42 %		∆=6.82 %	
SDC (mm:ss)	1.62 ± .2	1.4 ± .04^$^	1.44 ± .07	1.33 ± .05^#^	1.58 ± .22	1.29 ± .05^$^	1.33 ± .42	1.66 ± .28
	∆=13.5 %		∆=7.63 %		∆=18.3 %		∆=24.81 %	
Leg Tuck (reps)	2.4 ± .51	4.4 ± 2.36*	5.6 ± 3.86	8.2 ± 4.91^#^	3.2 ± 2.14	5.6 ± 2.45^#^	9.2 ± 6.74	11 ± 6.46
	∆=83.3 %		∆=46.42 %		∆=75 %		∆=19.56 %	
2MR (mm:ss)	17.45 ± 1.09	16.19 ± 1.1^#^	15.76 ± 1.05	15.34 ± .91^$^	17.13 ± 1.27	16.16 ± 1.29^$^	17.13 ± 1.27	16.41 ± .75
	∆=7.2 %		∆=2.66 %		∆=5.6 %		∆=4.20 %	
Total score	429.6 ± 7	466.8 ± 13.5^$^	467.8 ± 35.1	496.4 ± 25.2^$^	443.6 ± 20.7	497.2 ± 20.2^$^	480.6 ± 42.5	479.0 ±
	∆=8.6 %		∆=6.1 %		∆=12.0 %		∆=0.3 %	
Irisin (mcg/ml)	5.1 ± 0.5	7.2 ± 0.5^$^	4.8 ± 0.3	6.3 ± 0.6^$^	4.8 ± 0.3	7.6 ± 0.43^$^	5.1 ± 0.4	5.07 ± 0.4
	∆=41.1 %		∆=31.2 %		∆=58.3 %		∆=0.5 %	

Abbreviations: kg: Kilograms, BMI: body mass index, cm^2^: square centimeter, BFP: body fat percentage, VFI: Visceral Fat Index, LBM: Lean Body Mass, rep: repetition, 3-RM: 3 repetition maximum, SPT: Standing Power Throw, SDC: Sprint-Drag-Carry, 2MR: 2-mile Run, mm: ss: minute: second, mcg/ml: micrograms per milliliter, TAB: Tabata Training, COMB: Tabata Training+Cinnamon supplementation, CIN, Cinnamon supplementation, CON: Control.

*#$: compared with pre-test; * *p *< .05, ^#^ *p *< .01, ^$^ *p *< .001.

**Table 4.** Results of MANCOVA analysis between groups.

		Sum of squares	df	Mean square	F	*P-*value	Partial eta squared
Body mass (kg)	Between groups	32.7	3	10.9	.66	.05*	.047
	Within groups	379.3	40	16.4			
BMI (kg.m^−2^)	Between groups	8.7	3	2.9	1.2	.032*	.083
	Within groups	54	40	2.3			
BFP (%)	Between groups	25.2	3	8.4	11.6	.000^$^	.465
	Within groups	1.5	40	.072			
VFI (cm^2^)	Between groups	17.6	3	5.8	35.15	.000^$^	.725
	Within groups	3.4	40	.165			
LBM (kg)	Between groups	14.2	3	4.7	5.69	.005^#^	.299
	Within groups	17.3	40	.825			
3-RM Deadlift (kg)	Between groups	12.8	3	4.2	3.5	.031*	.208
	Within groups	25.2	40	1.2			
SPT (meter)	Between groups	280.8	3	93.6	8.0	.001^$^	.375
	Within groups	245.6	40	11.6			
Push-ups (reps)	Between groups	470.9	3	156.9	32	.000^$^	.706
	Within groups	103.7	40	4.9			
SDC (mm:ss)	Between groups	851.2	3	283.7	74.6	.000^$^	.848
	Within groups	80.2	40	3.8			
Leg Tuck (reps)	Between groups	63.5	3	21.1	1.95	.152	.128
	Within groups	227.9	40	10.8			
2MR (mm:ss)	Between groups	121.8	3	40.6	6.4	.003^#^	.324
	Within groups	133.2	40	6.3			
Total score	Between groups	6456.7	3	2152.2	107.6	.000^$^	.890
	Within groups	420.2	40	20.0			
Irisin (mcg/ml)	Between groups	11.4	3	3.8	22.35	.000^$^	.626
	Within groups	3.5	40	.170			

Abbreviations: kg: Kilograms, BMI: body mass index, cm^2^: square centimeter, BFP: body fat percentage, VFI: visceral fat index, LBM: lean body mass, rep: repetition, 3-RM: 3 repetition maximum, SPT: standing power throw, SDC: Sprint-Drag-Carry, 2MR: 2-mile run, mm: ss: minute: second, mcg/ml: micrograms per milliliter.

*#$: compared with pre-test; *p < .05, ^#^ *p *< .01, ^$^ *p *< .001.

**Figure 3.**
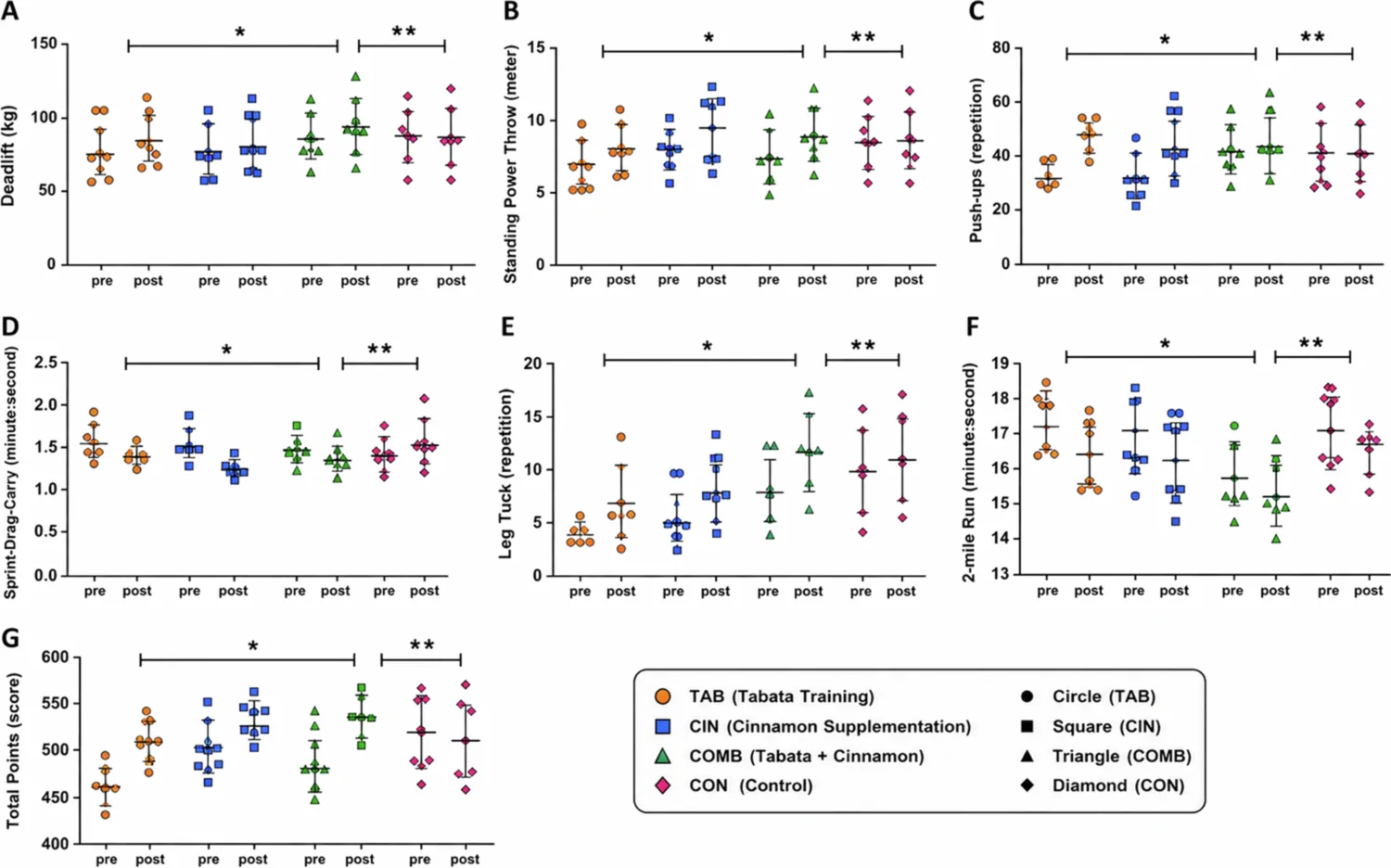
The changes in the ACFT test are shown in Figure 2 (A–G). TAB: Tabata training, COMB: Tabata training+cinnamon supplementation, CIN: Cinnamon supplementation, CON: Control. *Significant difference between TAB and CON groups, **Significant difference between COMB and CON groups (*P *< 0.05).

### 3.5. Serum irisin concentration

The changes in serum irisin concentration are shown in Figure 4-A. Serum irisin levels increased significantly in the COMB group from 4.83 ± 0.35 to 7.65 ± 0.43 μg/mL (*p *< 0.001), corresponding to a+58% elevation. The TAB group showed a moderate increase (+40%; *p *< 0.001), while the CIN group exhibited a smaller but significant rise (+29.5%; *p *< 0.001). No significant change was detected in the CON group (*p*=0.61). Between-group comparisons confirmed that the COMB group demonstrated a significantly greater increase in irisin than the other groups (*p *< 0.01), indicating that the combined intervention was associated with a greater irisin response than either intervention alone (Tables 3 and 4).

#### 3.5.1. Body composition

The changes in body composition and anthropometry are shown in Figure 4 (B-D) (Tables 2 and 3). There were significant group × time interactions for body weight, BMI, percent body fat, visceral fat index, and lean body mass (*p *< 0.001 for all). Post hoc analysis revealed significant reductions in body mass in all intervention groups compared to control (TAB: *p *< 0.01; CIN: *p *< 0.01; COMB: −7.5%, *p *< 0.05). Similar patterns were observed for BMI reductions (TAB and CIN: *p *< 0.05; COMB: −16.5%, *p *< 0.05). Percent body fat showed marked improvements, particularly in the combined group (TAB: −13%, *p *< 0.001; CIN: *p *< 0.001; COMB: −18.8%, *p *< 0.001). The visceral fat index decreased significantly (TAB: −20%, CIN: *p *< 0.001; COMB: −27.3%, *p *< 0.001). Lean body mass significantly increased in all intervention groups (TAB: *p *< 0.001; CIN: *p *< 0.001; COMB: +15%, *p *< 0.001) (Tables 3 and 4).

**Figure 4.**
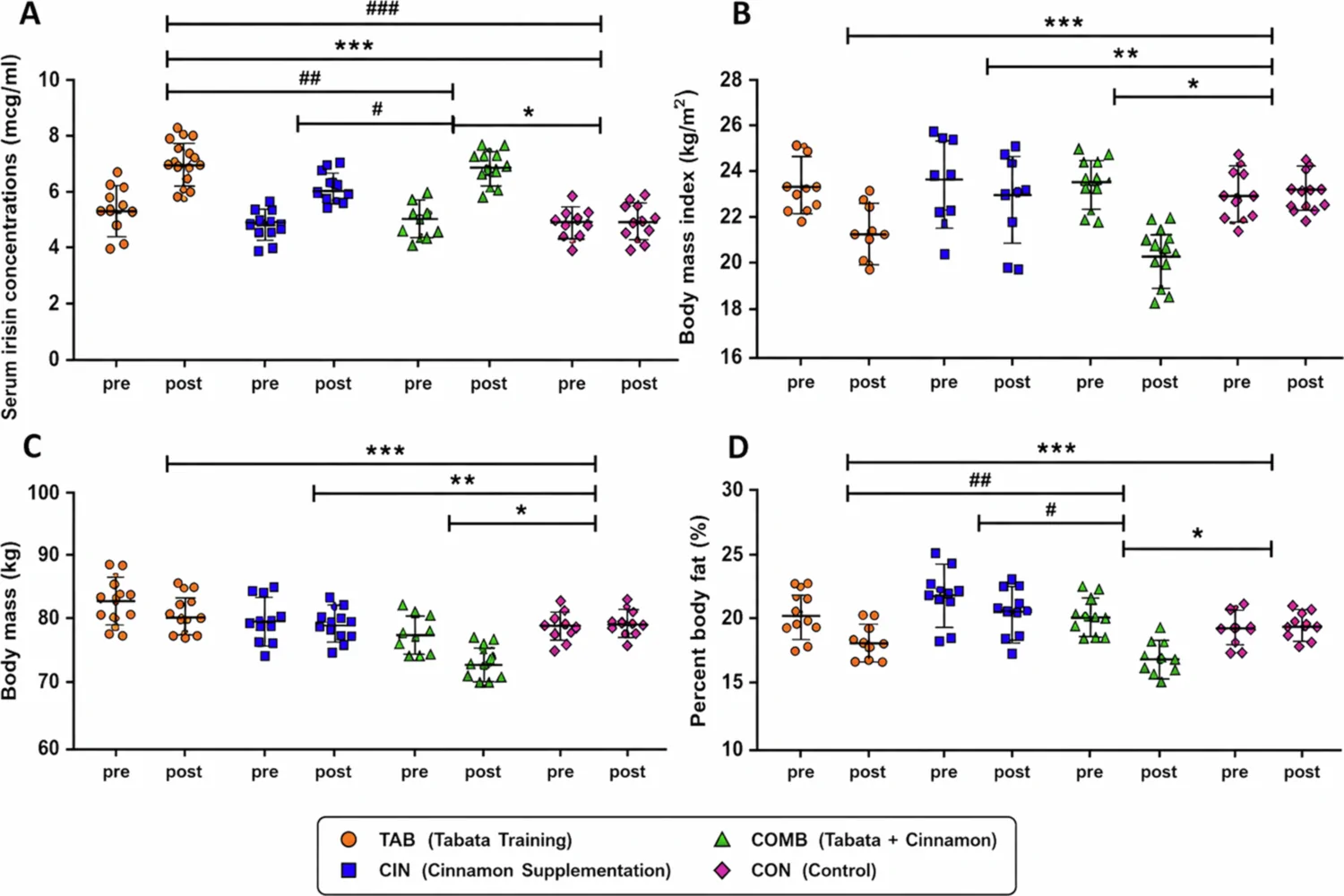
Effects of 6 weeks of interventions on (A) serum irisin concentrations (mcg/mL), (B) body mass index (BMI), (C) body mass, and (D) percent body fat. Data are presented as individual values with mean ± SD. TAB: Tabata training; CIN: Cinnamon supplementation; COMB: Tabata training plus cinnamon supplementation; CON: Control. *p *< 0.05, *p *< 0.01, *p *< 0.001 indicate significant between-group differences. ^#^ *p *< 0.05, ^##^ *p *< 0.01, ^###^ *p *< 0.001 indicate significant group × time interaction effects.

## 4. Discussion

This study explored the short-term effects of a 6-week Tabata exercise intervention combined with cinnamon supplementation on serum irisin levels, body composition, and functional performance. To establish a clear baseline for these interventions, it is important to first address the status of the control group. The control group consisted of active military cadets who did not receive the Tabata training intervention or cinnamon supplementation, nor were they administered a placebo (i.e. a no-placebo control group). Throughout the 6-week study period, these cadets did not participate in any structured high-intensity exercise program beyond their mandatory, standardized military training. This standard training included routine military drills, low-to-moderate intensity physical conditioning (e.g. formations, marching, and standard calisthenics), and daily military tasks required by their academy curriculum, which remained constant and identical across all study groups.

Significant improvements in body composition variables – such as BM, BMI, LBM, BFP, and VFI – were observed in the Tabata training intervention group. This suggests that Tabata training can play an effective role in enhancing body re-composition [32,33]. Past studies have shown that Tabata training has the potential to increase VO_2_max and anaerobic threshold in a short time frame of up to 4 weeks, largely due to mitochondrial biogenesis, increased oxidative capacity of the muscles, and neuromuscular recruitment [34]. The study identified notable improvements in ACFT test performance among male cadets after implementing the Tabata training protocol. It seems that Tabata training enhances both anaerobic and aerobic energy systems, leading to improvements in strength, endurance, and motor skill efficiency [35]. Although less than the maximal HIIT volume, a similar 3-week training program has been reported to increase VO_2_max and other tactical fitness outcomes, including in ROTC cadets who took a 6-week leave during training [36]. In addition, tabata-style training has been shown to achieve comparable or better results than traditional endurance training with much less time investment and is therefore quite compatible with the prevailing operating conditions [37].

Interestingly, the results from the combination group indicate that a greater improvement in ACFT performance was observed in those tests that combine muscle endurance and anaerobic power, i.e. sprint-drag-carry and hand-release push-up tests. These results are likely due to the very high neuromuscular recruitment and lactate tolerance associated with the Tabata regimen. The additional benefit observed in the cinnamon group may be due to reduced systemic inflammation and increased glycogen synthesis, which are attributed to cinnamon's antioxidant and insulin mimetic effects [38]. In summary, Tabata training effectively enhances functional performance, and the inclusion of cinnamon supplementation may provide additional benefits that extend beyond those achieved through training alone.

The most striking finding is that circulating levels of irisin increased dramatically in the COMB group. Myokines, such as irisin, secreted in response to physical activity, are thought to contribute to insulin sensitivity by promoting the browning of white adipose tissue, thermogenesis, and the metabolism of lipids and glucose [19]. Although HIIT has been shown to increase irisin [23], our findings suggest that cinnamon may increase this exercise-dependent response, possibly by stimulation of both PGC-1 alpha and FNDC5 alpha downstream. This is the first human study to demonstrate these concurrent effects of phytochemical supplements and HIIT on serum irisin in military personnel.

Cinnamon alone produced only minor changes in the visceral fat index and irisin, in line with reported insulin sensitization, lipolytic, and thermogenic effects [39,40]. Cinnamon has been shown to reverse obesity by activating AMPK signaling, altering GLUT4 expression, and reducing proinflammatory cytokines by increasing fat metabolism and metabolic flexibility [16,17]. It is particularly noteworthy that the body fat and BMI percentages decreased to a much lower extent in the group receiving both cinnamon and COMB compared to those receiving only TAB or CIN.

Cinnamon supplementation has been shown to have potential benefits on glucose homeostasis, fat oxidation, and lipid profiles in clinical and sports subjects [13]. Limited studies have been carried out on its use in trained individuals or under the guidance of systematic training programs. Our research fills this gap by providing early indications that the metabolic benefits observed with cinnamon supplementation may be enhanced when combined with high-intensity interval training. This combined response may be attributed to the convergence of cellular pathways, particularly those involving AMPK and irisin signaling [41,42]. A number of recent scientific reports suggest that irisin may be a mediator of the metabolic benefits resulting from exercise, but its susceptibility to various types of training and dietary supplements is unknown. Past studies have shown that chronic resistance or endurance training may increase irisin levels only slightly, but the results were not congruent [21,43].

The significant increase in irisin levels in the COMB group in our study suggests that the combined activation of myogenic pathways through HIIT and metabolic pathways influenced by phytochemicals may contribute to greater irisin release. This hypothesis is supported by animal models where polyphenol compounds have been shown to increase the expression of UCP1, FNDC5, and PGC-1 alpha, which are key mediators of browning and thermogenesis [44,45].

The findings of this study indicate that a Tabata training program combined with cinnamon supplementation leads to measurable improvements in physical performance, body composition, and metabolic markers among young, healthy male cadets. These results suggest that time-efficient, high-intensity interval training, when paired with a well-tolerated herbal supplement such as cinnamon, can be an effective and low-risk strategy for enhancing military fitness. Given the demands of military service and the importance of operational readiness, incorporating such interventions into routine training regimens may provide an effective way to optimize physiological capacity while minimizing resource and time burdens. However, further research is needed to confirm the long-term efficacy and safety of this combined approach across diverse military populations.

### 4.1. Limitations

Several limitations of the present study should be acknowledged. First, the intervention duration was relatively short (6 weeks), which limits conclusions regarding the long-term sustainability and durability of the observed physiological and performance adaptations. Second, the study sample consisted exclusively of young, healthy male military cadets; therefore, the findings may not be generalizable to female personnel, older individuals, or populations with metabolic or clinical conditions.

Third, body composition was assessed using multi-frequency BIA, which, although practical and suitable for field-based military settings, is less precise than gold-standard methods such as dual-energy X-ray absorptiometry or magnetic resonance imaging and may underestimate visceral adiposity while overestimating lean mass. In addition, hydration status was not objectively verified using urine specific gravity or plasma osmolality. While standardized pre-testing procedures were implemented (morning assessments following an overnight fast and avoidance of strenuous exercise, caffeine, and fluid intake), unmeasured variations in hydration may have influenced BIA-derived estimates.

Fourth, the study did not employ a placebo-controlled or double-blinded supplementation design, which limits the ability to fully exclude expectancy or behavioral effects related to cinnamon intake. Moreover, although participants were instructed to maintain their habitual diets and dietary intake was recorded, nutritional intake was not strictly controlled, which may have contributed to inter-individual variability in metabolic and body composition responses.

Finally, the investigation focused on a limited number of circulating biomarkers. The absence of additional inflammatory, hormonal, and metabolic mediators (e.g. insulin, IL-6, TNF-*α*, and cortisol) restricts the mechanistic interpretation of the observed changes. Accordingly, while the combined intervention appeared to produce greater improvements in several outcomes, these effects should be interpreted as potential or additive rather than definitively synergistic.

Future research should evaluate longer intervention periods, incorporate placebo-controlled designs, include diverse populations, and apply gold-standard body composition techniques alongside objective hydration assessment. Further investigation into dose‒response relationships, interactions with different exercise modalities, and underlying molecular mechanisms, including broader biomarker profiling, microbiome analysis, and functional imaging, would help clarify the physiological pathways involved.

## 5. Conclusions

Participation in a 6-week Tabata regimen produced significant improvements in both body composition and performance among young male military cadets, and when combined with cinnamon supplementation, it was associated with a greater increase in circulating irisin levels than either intervention alone. This elevation in irisin following the intervention may contribute to multiple beneficial metabolic effects and was accompanied by meaningful reductions in overall body weight and body fat. Collectively, these changes translated into substantial gains in functional capacity over a relatively short timeframe, underscoring the potential of integrating high-intensity intermittent exercise with targeted nutritional strategies to enhance physiological and metabolic outcomes.

These findings indicate a novel, practical, additive benefit to evidence-based exercise recommendations and dietary supplements. The application of such a protocol to military or tactical populations could improve physical readiness, injury risk mitigation, and long-term health resilience to an operational stress state. Notably, the intervention is short in duration and low in cost, which implies that it is possible to implement it on a large scale, both in institutions and in the field. This is the first randomized controlled trial to prove that cinnamon supplementation can enhance the physiological response of high-intensity functional training, in terms of irisin release and composite functional performance, to our knowledge. It is necessary to study the longevity, the mechanism, and the applicability of these results regarding different genders, ages, and areas of performance in further research. In short, combining plant-based nutritional strategies with time-efficient HIIT training programs may represent a practical approach for improving metabolic health and physical performance among tactical populations.

## Data Availability

The data that support the findings of this study are available from, but restrictions apply to the availability of these data, which were used under license for the current study, and so are not publicly available. Data are, however, available from the corresponding author upon reasonable request.
